# Proteomic analysis reveals diverse proline hydroxylation-mediated oxygen-sensing cellular pathways in cancer cells

**DOI:** 10.18632/oncotarget.12632

**Published:** 2016-10-13

**Authors:** Tong Zhou, Luke Erber, Bing Liu, Yankun Gao, Hai-Bin Ruan, Yue Chen

**Affiliations:** ^1^ Department of Biochemistry, Molecular Biology and Biophysics, University of Minnesota at Twin Cities, Minneapolis, MN 55455, USA; ^2^ Department of Integrative Biology and Physiology, University of Minnesota Medical School, Minneapolis, MN 55455, USA

**Keywords:** proline hydroxylation, oxygen-sensing, posttranslational modification, hypoxia, LCMS

## Abstract

Proline hydroxylation is a critical cellular mechanism regulating oxygen-response pathways in tumor initiation and progression. Yet, its substrate diversity and functions remain largely unknown. Here, we report a system-wide analysis to characterize proline hydroxylation substrates in cancer cells using an immunoaffinity-purification assisted proteomics strategy. We identified 562 sites from 272 proteins in HeLa cells. Bioinformatic analysis revealed that proline hydroxylation substrates are significantly enriched with mRNA processing and stress-response cellular pathways with canonical and diverse flanking sequence motifs. Structural analysis indicates a significant enrichment of proline hydroxylation participating in the secondary structure of substrate proteins. Our study identified and validated Brd4, a key transcription factor, as a novel proline hydroxylation substrate. Functional analysis showed that the inhibition of proline hydroxylation pathway significantly reduced the proline hydroxylation abundance on Brd4 and affected Brd4-mediated transcriptional activity as well as cell proliferation in AML leukemia cells. Taken together, our study identified a broad regulatory role of proline hydroxylation in cellular oxygen-sensing pathways and revealed potentially new targets that dynamically respond to hypoxia microenvironment in tumor cells.

## INTRODUCTION

Proline hydroxylation (Hyp) is a key oxygen-sensing posttranslational modification that is dynamically modulated during tumor cell proliferation and angiogenesis [1–6]. The modification is mediated by the evolutionarily conserved prolyl hydroxyalses that add a single oxygen to the carbon on the proline pyrrolidine ring and form an (*S)*-configured hydroxyl group at the gamma position. O_2_-dependent nature of hydroxylation reaction renders prolyl hydroxylases as key oxygen sensors that are dynamically responsive to the hypoxia microenvironment of the cells or organisms. The modification subtly affects the protein structures, activities and the properties of protein-protein interactions in the cell and plays a pivotal role in cancer development and disease progression [7–11].

The most well-known proline hydroxylation substrate is collagen, an essential structural molecule to support cell matrix and skeleton structures [12–15]. The hydroxylation on the conserved PG motif is required for the proper folding of the collagen polypeptide chain and the release of mature collagen proteins from endoplasmic reticulum. Proline hydroxylation is also known to regulate hypoxia-inducible-factor-1 alpha (HIF-1α) protein, an important transcription factor [11]. Under normoxia condition, hydroxylated HIF-1α protein is specifically recognized by von Hippel-Lindau (pVHL) E3 ubiquitin ligase, which leads to rapid HIF-1α protein degradation [2]. Hypoxia microenvironment, on the other hand, reduces the hydroxylation abundance on HIF-1α and prevents its rapid degradation. Stabilized HIF-1α leads to transcriptional activation of nearly 100 proteins in the key hypoxia-response cellular pathways, which are critical for cancer cell survival under low O_2_ environment [11]. Recent biochemical studies with high resolution mass spectrometry have identified and validated a number of other proline hydroxylation substrates including FOXO3a, Argonaute 2, ATF-4 and PKM [16–22], which revealed diverse HIF-independent oxygen-sensing activities in RNA interference and transcriptional regulation. These evidence suggest that the hypoxia-response mechanism mediated through proline hydroxylation regulates diverse cellular pathways and signaling processes in cells and may play a much wider role in regulating cellular physiology and protein functions.

Despite these advances, only limited number of proline hydroxylation substrates have been identified and validated in cancer cells, which hinder the effort to fully understand the cellular response to the hypoxia microenvironment. To address this challenge, we have developed an immunoaffinity-purification assisted approach to system-wide identify proline hydroxylation substrates in cancer cells. Our analysis revealed a broad range of Hyp substrate proteins and essential cellular pathways targeted by this important protein modification.

## RESULTS

### System-wide analysis of proline hydroxylation proteome

**Figure 1:**
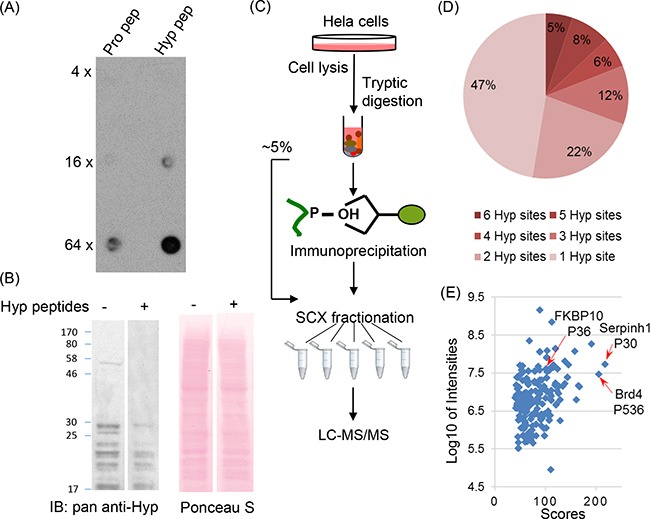
Systematic analysis of proline hydroxylation proteome with immunoaffinity purification and exhaustive LC-MS/MS analysis **A.** Dot-blot assay for antibody specificity validation. Pro- and Hyp-containing peptide libraries were dotted on the nitrocellulose membrane with 0.125 μg, 0.5 μg and 2 μg on each dot from top to bottom, respectively. Pan-anti Hyp antibody was incubated with the membrane for two hours at room temperature prior to detection. **B.** Competition assay. Pan-anti Hyp antibody was incubated with Hyp-containing peptide library for 16 hours at 4°C. Western blotting of two lanes with equal amounts of Hela whole cell lysate was performed with pan-anti Hyp antibody with or without prior incubations with Hyp peptides. **C.** Schematic diagram of the proteomics workflow. Hela cells were lysed and proteins were digested by trypsin. Peptides were subject to immunoaffinity purification and exhaustive LC-MS/MS analysis with fractionation for identification. **D.** Distribution of the number of Hyp sites identified per peptide. Over 30% of the peptides were identified with more than two Hyp sites. **E.** The scatterplot of Hyp peptide identifications with Maxquant scores and intensities.

We first developed and validated a pan-antibody recognizing peptides containing trans-4-hydroxylated proline (Figure 1A–1B, Supplementary Figure S1). Using this antibody, we performed an initial proteomic analysis to systematically identify proline hydroxylation targets in HeLa cells (Figure 1C, Supplementary Figure S2). The cells were lysed following a standard protocol, and the proteins were digested by trypsin into short peptides. The peptides were subject to immunoaffinity purification using the custom-made antibody to enrich peptides bearing proline hydroxylation. Peptides with or without immunoaffinity enrichment were then fractionated using strong-cation exchange chromatography into 6 fractions and analyzed by nano-HPLC/MS/MS for deep proteomic analysis to identify hydroxyproline-containing peptide substrates. The LCMS data were processed by MaxQuant and Andromeda search engines for peptide and protein identifications. The database search against human UniProt sequences revealed over 1000 candidate proline hydroxylation sites. However, careful manual inspection of peptide-spectrum matches suggested that some of the identifications are ambiguous hits with incomplete peptide backbone fragmentation and questionable site localizations. In such cases, common chemical oxidation artifacts such as Met and Trp oxidation could confuse the search engine and erroneously assign the modification to the nearby Pro, which has been considered as a significant technical challenge in the confident identification of proline hydroxylation sites [22]. To address this issue, we developed an in-house script to analyze the MS/MS fragmentation patterns of peptide-spectrum matches and required that at least two distinct peptide fragment ions in each spectrum must be annotated to confidently assign the mass shift to Pro comparing to the nearest Met and Trp amino acids at both peptide N-terminal and C-terminal sides (see Materials and Methods). Although such filtering also removed some potentially true proline hydroxylated peptides, it significantly improved the confidence and quality of the dataset, which are essential for the bioinformatics analysis of the proline hydroxylation proteome. After careful screening, we identified 562 proline hydroxylation sites from 272 proteins in HeLa cells (Supplementary Table S1). Nearly 20% Hyp peptides contain at least four hydroxylation sites (Figure 1D). About one-third of the sites were identified only by immunoaffinity purification, suggesting that the application of this strategy contributed to the complementary coverage for Hyp peptide identification. Our results identified many previously well-known proline hydroxylation targets including collagen, actin and FKBP10 proteins [22–25], while at the same time, the majority of proline hydroxylation substrates have not been reported before (Figure 1E). The dataset provides a rich resource to reveal the diverse cellular pathways involved in proline hydroxylation-mediated oxygen sensing mechanisms in cancer cells.

### Flanking sequence and secondary structural preference of proline hydroxylation sites

We first performed flanking sequence analysis using all +/− 15 amino acids in protein sequences surrounding the proline hydroxylation sites. The data showed that proline is one of the most frequently appearing amino acids surrounding hydroxylated proline, suggesting that many of the Hyp modifications occur in proline-rich sequence regions. Interestingly, we found 59% of Hyp peptides identified in our dataset bearing more than two Hyp modifications per peptide (Figure 1D) and such percentage is much higher than we typically observe in lysine acetylation proteomics analysis. We notice that amino acids with short side chains such as Gly and Ala appear very frequently in the immediate vicinity of hydroxylated proline. This observation agrees well with the knowledge that proline hydroxylation sites on collagen are often followed by Gly to form PG motifs. Our dataset revealed 65 sites sharing common PG motifs, suggesting that the enzyme, prolyl-4-hydroxylases that regulate collagen proline hydroxylation, may regulate the hydroxylation on diverse protein substrates. Motif enrichment analysis using Motif-x program [26] identified PP, PxP and PGxP as highly enriched motifs among proline hydroxylation sites (Supplementary Figure S3).

Proline hydroxylation is known to be essential for stabilizing collagen secondary structures. We want to ask whether the localization of identified proline hydroxylation sites have strong association with substrate protein's secondary structures. Towards this goal, we extracted the manually-curated UniProt secondary structural information for each identified proline hydroxylation sites and for all the proline sites in each Hyp substrate proteins. We found 217 Hyp sites whose proteins have UniProt annotated structural information. Surprisingly, among these sites, 134 proline hydroxylation sites (62%) are located in one of the annotated secondary structures (STRAND, HELIX and TURN), while in the corresponding proteins, only 6% of total proline residues locate in one of these secondary structures. When considering all amino acids, only 18% were involved in the secondary structure among these proteins. The lower percentage of Pro sites participated in the secondary protein structural is expected and agree well with previous knowledge on the characters of proline imino peptide backbone [27, 28]. The significant enrichment of Hyp sites locating in the secondary structure comparing to the distribution of Pro (HyperG test P<1.6×10^−108^) and total amino acids (HyperG test P<7.5×10^−47^), suggest that proline hydroxylation likely specifically targets those Pro sites involved in the protein secondary structure and may have important contribution to the protein folding and structural stability for diverse substrate proteins.

**Figure 2:**
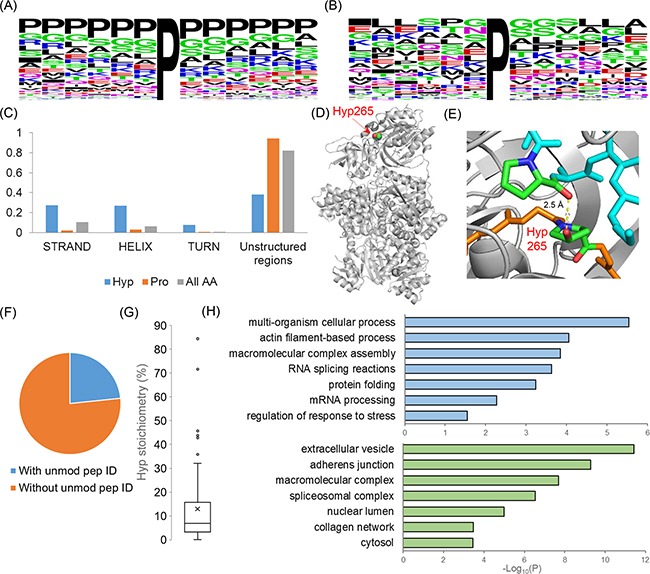
Analysis of the structural and functional characteristics of proline hydroxylation proteome in Hela cells **A.** Flanking sequence distributions of proline hydroxylation sites identified in this study. **B.** Flanking sequence distributions of proline hydroxylation sites from peptides identified with less than three Hyp sites. Flanking sequences were visualized with WebLogo [81]. **C.** The distribution of the secondary structures of proline hydroxylation sites (blue bars) comparing to proline (orange bars) and all amino acids (grey bars). **D.** and **E.** structural illustrations showing that the hydroxylation of Pro265 on CPS1 may form a hydrogen bond with the alpha-oxygen of the nearby Pro87 (PDB: 5DOU). **F.** The percentage distribution of Hyp peptides identified with and without the identification of the unmodified counterparts. **G.** The distribution of the calculated stoichiometries of Hyp sites with an average stoichiometry of 13%. **H.** Gene ontology annotation analysis of proline hydroxylation proteome with statistical enrichment in biological processes (upper panel) and cellular compartments (lower panel) (BH corrected P<0.05).

Carbamoyl phosphate synthase (CPS1) is a rate-limiting enzyme in the urea biosynthesis and ammonia metabolic pathways. In our dataset, we identified CPS1 as a novel proline hydroxylation substrates with hydroxylation on Pro265 that locates within the glutamine amidotransferase domain of CPS1. Using structural analysis, we showed that hydroxylation on Pro265 may form a strong hydrogen bond between the Pro265 hydroxyl group and the alpha carboxyl group of Pro87 at the neighboring peptide backbone, potentially affecting the structural stability or enzymatic function of the protein (Figure 2D–2E).

### Stoichiometric analysis of proline hydroxylation substrates

Site-specific PTM stoichiometry is a key metric to quantitatively understand the physiological importance of individual protein modification sites to the substrate protein's function and activity. To systematically estimate proline hydroxylation site stoichiometry, we extracted all sites identified by the direct LCMS analysis without enrichment and compared the precursor ion intensities of both modified and unmodified peptides. Interestingly, over three-quarters of these proline hydroxylated peptides were identified only in modified form without the identification of the corresponding unmodified counterpart (Figure 2F), which include all the poly-proline hydroxylated peptides (the peptides with more than two sites per peptide). Site-specific stoichiometries were successfully calculated for seventy-two proline hydroxylation sites using the summed precursor ion intensities for the pair of modified and unmodified peptides for each site (Figure 2G and Supplementary Table S2). Our data showed an average stoichiometry of proline hydroxylation about 13%, with nearly 20% percent of sites having more than 20% stoichiometry. Comparing to recently published data, proline hydroxylation has much higher stoichiometry than lysine acetylation [29–31]. However, considering a large portion (>75%) of Hyp sites not quantifiable without the identification of unmodified counterpart including the poly-Hyp peptides from collagen (Figure 2F) and *a priori* knowledge that collagen hydroxylation is essential for its structure and function [8, 12, 32, 33], it is likely that the average of 13% proline hydroxylation stoichiometry based on quantifiable sites significantly under-estimated the overall proline hydroxylation abundance in cells.

### Functional annotation analysis of proline hydroxylation proteome

To systematically study the physiological significance of proline hydroxylation substrates, we performed pathway enrichment analysis with Gene Ontology annotation analysis. Our results showed that proline hydroxylation substrates are highly enriched in the biological processes including multi-organism processes (adj P=2.9×10^−6^), macromolecular assembly (adj P=1.4×10^−4^), RNA splicing (adj P=2.4×10^−4^) and regulation of response to stress (adj P=2.8×10^−2^) (Figure 2H upper panel). In cellular compartment enrichment analysis, we found that proline hydroxylation substrates are widespread across different compartments but have the most apparent enrichment in extracellular vesicle (adj P=3.6×10^−12^), macromolecular complex (adj P=2.0×10^−8^), nuclear lumen (adj P=1.0×10^−5^) and cytosol (adj P=3.6×10^−4^) (Figure 2H lower panel). Gene classification by PANTHER system [34] showed cytosol proteins account for nearly half of the total Hyp proteome (~42%) while ~28% of Hyp proteome is from the cell organelles which mostly consist of cytoskeleton (~43%) and nucleus (~43%) proteins (Supplementary Figure S4). Few mitochondria proteins were identified to be proline hydroxylated, which agrees well with the current knowledge and cellular compartment annotations of prolyl hydroxylases mostly in the cytosol and nucleus [35–37], though it remains likely that mitochondria protein proline hydroxylation may be mediated by the radical-induced mechanism as previously suggested [38].

**Figure 3:**
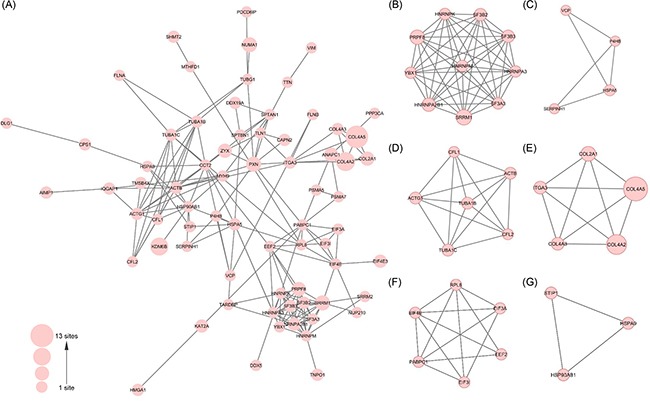
Protein interaction network analysis of proline hydroxylation proteome in Hela cells **A.** The interaction network of proline hydroxylation proteins identified in this study and **B-G.** an example list of the sub-networks of proline hydroxylation proteins with high interconnectivity.

To investigate how proline hydroxylation substrates involve in the macromolecular assembly, we performed protein complex enrichment analysis with manually-curated CORUM database. We found that proline hydroxylation substrates were highly enriched in over 80 protein complexes, including spliceosome (P=6.9×10^−10^), 60s APC containing complex (P=8.0×10^−5^), EIF3 core complex (P=1.1×10^−3^) and 20S proteasome (P=1.4×10^−2^) (Supplementary Table S3). Analysis of proline hydroxylation proteome with protein-protein interaction database STRING showed that Hyp substrates formed vast and highly connected interaction networks (Figure 3A). Using subnetwork connectivity analysis, we identified several representative subnetworks including RNA spliceosome complex, cytoskeleton tubulin and collagen interaction networks (Figure 3B–3G).

Using online disease association database and annotation enrichment tool [39, 40], we performed disease enrichment analysis. Our data revealed the significant enrichment of proline hydroxylation substrates in stress and shock associated cellular pathways (adj P=1.3×10^−8^) as well as diseases associated with viral infections such as HIV (adj P=7.1×10^−6^). The hydroxylation substrate proteins identified in this pathway include heat shock proteins (HSP90AA1, HSP90AB1, HSPA5), NUAK family kinase (NUAK2), stress-induced phosphoprotein (STIP1) (Supplementary Table S4). The data suggested that proline hydroxylation may directly involve in regulating key protein activities in the stress-response cellular pathways, which are critical to cellular survival and function under stress conditions.

### Validation of proline hydroxylation substrates with synthetic peptides

**Figure 4:**
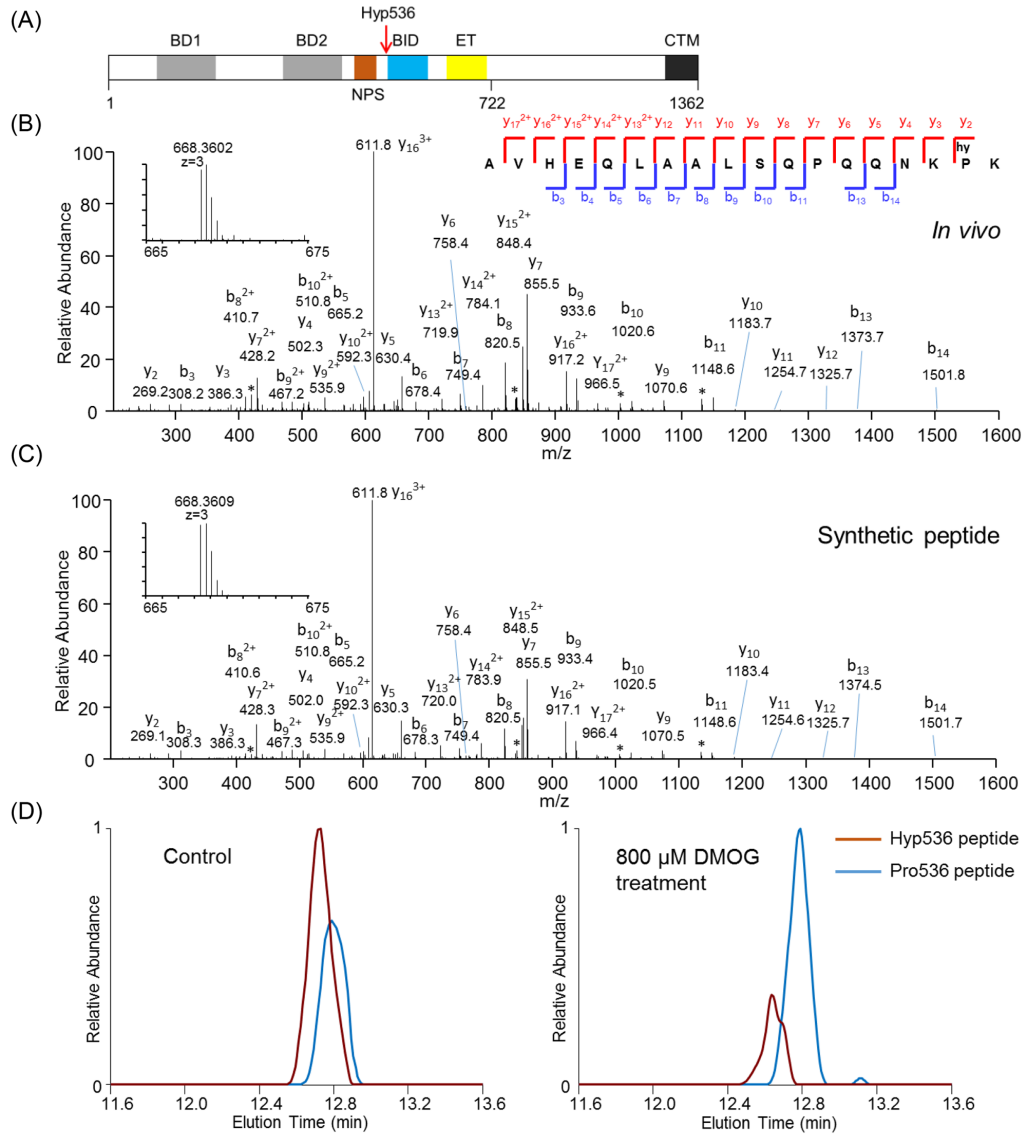
Identification and validation of Brd4 proline hydroxylation at Pro536 **A.** Brd4 proline hydroxylation Hyp536 locates at the junction between NPS and BID domain. **B.** in vivo and **C.** synthetic peptide fragmentation and precursor ion mass spectra comparison. “b” and “y” ions designate peptide backbone fragment ions containing peptide N- and C-terminus respectively. **D.** HPLC elution profile analysis of the Brd4 peptides bearing Hyp536 (red line) and Pro536 (blue line) under control and 800 μM DMOG-treated conditions.

We performed validation experiments for 6 peptides using fragmentation of synthetic peptides bearing the same peptide sequences and modifications. These identifications included Brd4 (Uniprot: O60885) Hyp536 (Figure 4 and Supplementary Figure S5), histone H2B 2-E (UniProt: Q99879) Hyp51 (Supplementary Figure S6A), proteasome subunit alpha-5 (UniProt: P28066) Hyp222 (Supplementary Figure S6B) and alpha7 (UniProt: O14818) Hyp149 (Supplementary Figure S6C), Serpin H1 (UniProt: P50454) Hyp30 (Supplementary Figure S6D), and FKBP10 (UniProt: Q96AY3) Hyp36 (Supplementary Figure S6E). Comparison of the MS/MS spectra between the in vivo peptides and synthetic peptides showed excellent matches, suggesting the high confidence of the proteomic identification of Hyp targets.

### Functional analysis of Brd4 proline hydroxylation

Brd4 is a key transcription factor that has been recently identified to play an important role in leukemia cell proliferation [41–43]. In our dataset, we identified Brd4 as a novel Hyp substrate with Pro536 hydroxylation at the junction between phosphoserine-rich NPS domain and the lysine-rich BID domain (Figure 4A–4C). Recent studies showed that the interaction between phosphorylated NPS domain and BID domain is essential for full Brd4 transcriptional activity and interaction with chromatin-binding proteins [44].

To further understand the physiological significance of the proline hydroxylation on Brd4, we performed functional analysis. First, we asked whether the proline hydroxylation abundance on Brd4 is enzymatically regulated. We treated the cells with DMOG, a well-established inhibitor of prolyl hydroxylases, and applied parallel reaction monitoring (PRM) in mass spectrometry to monitor the relative abundance of the Brd4 Hyp peptide (containing Hyp536) and its corresponding unmodified counterpart in Hela cells (Figure 4D). The data showed that DMOG treatment, which inhibits prolyl hydroxylase activities and mimic hypoxia condition, led to significantly decreased prolyl hydroxylation abundance on Brd4 relative to its unmodified peptide (Hyp stoichiometry from 59% to 24%), suggesting that Brd4 proline hydroxylation is enzymatically regulated by prolyl hydroxylases.

**Figure 5:**
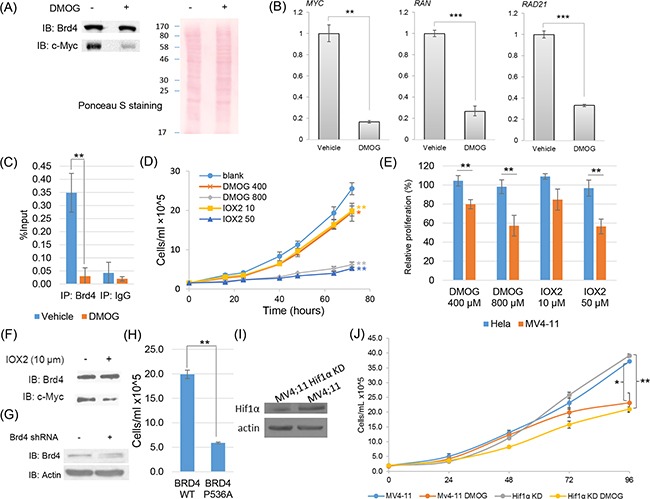
Functional characterization of proline hydroxylation pathway on Brd4 transcriptional activities and cell proliferation in MV4;11 cells **A.** Western blotting analysis showing that the DMOG treatment of MV4;11 cells (800 μM, 24 hrs) significantly decreased the protein abundance of c-Myc. **B.** Quantitative real-time PCR analysis showing that the DMOG treatment of MV4;11 cells significantly decreased the transcriptional expression of known Brd4-targeted genes. **C.** Chromatin immunoprecipitation and quantitative PCR (ChIP-qPCR) analysis of Brd4 binding to the c-Myc promoter in MV4;11 cells with or without DMOG treatment. **D.** Cell proliferation assay of MV4;11 cells under control, DMOG and IOX2 treatment. Statistical analysis was performed between treated and control cells at 72 hrs. **E.** The relative proliferation of Hela and MV4;11 cells under DMOG (400 μM, 800 μM) and IOX2 (10 μM, 50 μM) treatments for 24 hrs comparing to the vehicle-treated control. **F.** Western blotting analysis showing that IOX2 treatment (10 μM, 16 hours) significantly decreased the protein abundance of c-Myc. **G.** Cell proliferation assay of MV4;11 cells with shRNA knockdown of endogenous Brd4 and expression of WT Brd4 and Brd4 with P536A mutation. **H.** Western blotting analysis showing that HIF1α was significantly knocked down in a stable MV4;11 cell line. **G.** Cell proliferation assay demonstrating that the growth of MV4;11 cells with and without HIF1α knocked down were both strongly inhibited by DMOG treatment (400 μM). All statistical analysis were performed with three biological replicates (Student's t-test, * p<0.05, ** p<0.01).

Next, we want to ask whether the inhibition of proline hydroxylation impairs Brd4 transcriptional activity. We selected MV-4,11 cells as a model, which is a leukemia cell line highly dependent on Brd4 transcription activity and very sensitive to Brd4 inhibitor JQ1[45]. We performed qRT-PCR to monitor the transcription of several of known Brd4 transcriptional targets, c-Myc, Ran and Rad21[46, 47]. Our data showed that inhibition of prolyl hydroxylase activities using DMOG strongly reduced the abundance and transcriptional expression of Brd4 targets, suggesting that Brd4 transcriptional activity is potentially regulated by proline hydroxylation (Figure 5A–5B). To probe the possible mechanism of DMOG-regulated Brd4 transcription activity, we performed chromatin immunoprecipitation coupled with quantitative PCR (ChIP-qPCR) analysis and evaluated the dynamics of Brd4 chromatin binding under DMOG treatment in MV4;11 cells. Our data showed that DMOG treatment significantly reduced Brd4 binding to the c-Myc promoter in AML leukemia cells (Figure 5C). Since Brd4 transcriptional activity has been shown to be critical for AML cell proliferation and survival, we next asked whether the altered Brd4 transcription activity by prolyl hydroxylase inhibitors will affect leukemia cell growth. Towards this goal, we performed cell proliferation assay under the control and DMOG treatment condition. Our data showed that the growth of MV4;11 cells was significantly inhibited upon DMOG treatment, while the growth of the model cell line, Hela cell, is much less sensitive to DMOG treatment (Figure 5D–5E).

Since DMOG is a competitive inhibitor mimicking alpha-ketoglutarate (α-KG), it may have off-target effects on enzymes that require α-KG as cofactors other than prolyl hydroxylases. To address this issue, we repeated the assay with IOX2[48, 50], a recently developed compound that is highly specific to PHD prolyl hydroxylases comparing to other enzymes that require α-KG as co-factors. Our data confirmed that inhibition of prolyl hydroxylases by IOX2 also strongly reduced the expression of c-Myc in MV4;11 cells and inhibited leukemia cell growth (Figure 5D and 5F). These data demonstrated that the enzymatically catalyzed trans-4-hydroxyproline modification is essential for MV4;11 cell proliferation. To further demonstrate the site-specific role of proline hydroxylation on leukemia cell activity, we generated a Brd4 mutant plasmid with P536A through site-directed mutagenesis and identified an shRNA targeting 3′UTR to knock down the endogenous Brd4 (Figure 5G). Knockdown of Brd4 has been previously shown to significantly inhibit AML cell proliferation [43]. We found that expression of mutant Brd4 with the concurrent knockdown of endogenous Brd4 led to the significantly reduced MV4;11 cell proliferation comparing to the expression of the wild-type Brd4, suggesting that site-specific proline hydroxylation is critical for MV4;11 cell growth and function (Figure 5H).

Since the treatment of prolyl hydroxylase inhibitor is known to stabilize hypoxia-inducible factor1 alpha and previous studies showed that overexpression of HIF1α may lead to the growth arrest of leukemia cells [51], we wanted to ask whether the growth inhibitory phenotype induced by prolyl hydroxylase inhibitors is dependent on HIF1α. Towards this goal, we generated a stable MV-4;11 cell line with HIF-1α significant knockdown using lentivirus-mediated shRNA transfection (Figure 5I). With this cell line, we repeated the cell proliferation assay and our data showed that the growth inhibitory effect of leukemia cells by prolyl hydroxylase inhibitors is HIF-1α independent (Figure 5J).

## DISCUSSION

Proline hydroxylation is an ancient posttranslational modification first discovered in 1902 [52]. However, the enzymatic regulatory pathways of this modification were understood only recently [3–6, 12]. As an irreversible modification, the proline hydroxylation is catalyzed by prolyl hydroxylases which require oxygen as co-substrate and iron, alpha-ketoglutarate and ascorbate as co-factors. Such requirements intimately link the prevalence of proline hydroxylation with key cellular metabolic states including oxygen availability, iron concentration as well as energy homeostasis. In recent years, advances in high resolution mass spectrometry led to the identification of an increasing number of proline hydroxylation targets [16–22]. These recently identified substrates involved in the transcriptional activation or maintenance of diverse hypoxia-response cellular pathways that are critical for tumor initiation and proliferation, including angiogenesis, glycolysis, DNA damage response and inflammation. Despite such advances, the functional and substrate diversity of proline hydroxylation proteome in cancer cells remain largely unknown and the precise identification of proline hydroxylation sites still suffer from ambiguous site-localizations in the large-scale proteomics data analysis[22]. To address these challenges and fill in this important knowledge gap, we performed a systematic study to expand the proline hydroxylation proteome and applied stringent criteria for the identification and validation of candidate Hyp targets. We integrated immunoaffinity enrichment with exhaustive HPLC-mass spectrometry analysis and identified 562 proline hydroxylation sites on 272 protein substrates in HeLa cells. Other than collagen hydroxylation sites, most of the sites identified in our study have not been reported before, giving us a unique opportunity to study the functional diversity of proline hydroxylation proteome and its critical roles in cancer cell activities and survival.

Proline is a nonpolar, aliphatic amino acid with a unique secondary amine and a pyrrolidine side chain. The structural specifications give proline an exceptional conformational rigidity which can play characteristic roles in the secondary structure of a protein [53]. Proline is frequently found in turns and non-repetitive secondary protein structures [27]. It is a structural disruptor in alpha helix and beta sheet and usually exists at the ends of alpha helices and the edge of beta-sheets [28, 54]. Our analysis of annotated PDB structures showed that only 6% Pro tend to locate within the regular secondary structures, which is in accordance with previous studies, while surprisingly, 62% of proline hydroxylation with structural annotations identified in this study occur in the regular secondary structures, strongly suggesting that hydroxylation preferentially targets proline sites involved in the secondary structure and potentially promote the stabilization of the substrates’ protein structure. Mechanistically, hydroxylation of proline may promote the hydrophilic interactions and induce stereo electronic effect, which contribute to the stable helix in collagen. Studies have shown that proline hydroxylation may increase the residue's polarity and serve as a substitution in forming H-bond to compensate for the lack of hydrogen in the backbone nitrogen [33, 55]. In addition, proline hydroxylation induces characteristic stereoelectronic effect due to the electron-withdrawing ability of OH. The *gauche* effect and n-π* interaction can strengthen X-Hyp dihedral angels and stabilize helical structure [56]. Proline also plays an important role in regulating protein-protein interactions [57, 58]. Hydroxylation of the proline's hydrophobic pyrrollydine ring on the protein surface may fine-tune the surface polarity and potentially alter the properties of protein-protein interactions [59–61].

Analysis of flanking sequence of proline hydroxylation sites showed strong preference of the Hyp modification sites with Gly, Ala and poly-proline as neighboring amino acids. Indeed, nearly one-third of the tryptic peptides with proline hydroxylation (82/262) contain at least three Hyp modification sites. Such percentage is much higher than the typical analysis of phosphorylation and lysine acetylation proteome (data not shown). The preference of small non-polar amino acids like Gly as neighboring residues also agree well with the knowledge on proline hydroxylation sites on collagen, which is considered as a key motif for the prolyl-4-hydroxylase that mediates collagen proline hydroxylation. The prevalence of the peptides containing the PG motif suggests that prolyl-4-hydroxylase likely have more diverse targets than previously thought.

Cellular stress response pathways are strongly enriched with proline hydroxylation substrates, including key structural molecules and chaperone proteins. Such finding suggests that this oxygen-sensing modification could serve as a direct regulatory mechanism that mediates the activities of these pathways under hypoxia microenvironment. In addition, as trans-4-hydroxylproline plays a critical role in stabilizing trans-peptide bond and secondary protein structure, the lack of oxygen could strongly impact the folding and proper maintenance of diverse protein structures and the accumulation of incorrectly folded proteins may induce cellular stress responses. Indeed, unfolded protein response (UPR) in ER is one of the major hypoxia-induced cellular response pathways [57–60]. The hyperactivated UPR pathways are important for cancer cells to adapt to the hypoxia states and have become novel targets for cancer treatment. The identification of a wide range of structural and chaperone proteins with proline hydroxylation in stress-response pathways demonstrate a potentially critical role of proline hydroxylation in mediating protein folding and ER stress in cancer cells.

Messenger RNA processing and RNA splicing processes are some of the most enriched cellular pathways with proline hydroxylation substrates in Hela cells, which agrees with previous studies that have linked alternative RNA splicing and microRNA processing with hypoxia and oxygen sensing [20, 21, 66, 67]. Our data suggests a potentially critical role of proline hydroxylation pathways on the RNA processing in cancer cells. The identification and validation of proline hydroxylation site (Hyp50) on histone H2B further expands the inventory of histone marks. The location of the Hyp50 site at the junction between acetyllysine-rich N-terminal tail and the C-terminal histone globular domain indicates a potentially direct oxygen-dependent regulation of chromatin structure and epigenetic regulation. Despite the identification of novel Hyp targets, there are plenty of room to improve the proteomics analysis workflow through strategies with higher sensitivities and specificities. Comparing to the site-specific antibodies, we found that the custom-developed polyclonal pan-specific Hyp antibodies have varied affinities and specificities against sites with different flanking sequences and therefore limit its application in the Western blotting or the affinity enrichment of low abundance Hyp targets such as HIF1α. In addition, proline hydroxylation is known to promote the degradation of many substrates such as HIF1α, HIF2α, Foxo3a and ATF-4 [17–19], which were not identified in our initial proteomics screen. Inhibition of protein degradation pathways prior to the enrichment analysis may allow the identification of these important substrates with rapid turnover. We also acknowledge that confident identification and localization of proline hydroxylation sites through LCMS requires extensive and careful analysis of HPLC tandem mass spectrometry data. Issues like amino acid polymorphism, inefficient backbone fragmentation and co-eluting peptide interference may still lead to false-positive identifications. High-throughput sequencing and complex algorithms for LCMS analysis will be necessary to address these challenges.

Recently, bromodomain-containing proteins have emerged as novel targets for cancer treatments [41–43, 68]. Brd4 is one of the most well-studied bromodomain-containing proteins playing critical roles in c-Myc hyperactivation and leukemia cell proliferation. Our study identified Brd4 as a novel proline hydroxylation substrate with nearly 60% stoichiometry under normoxia states and its hydroxylation level is enzymatically regulated. Inhibition of prolyl hydroxylase activities significantly diminished the expression of Brd4-targeted genes as well as the proliferation of leukemia cells. Although the detail mechanisms of hydroxylase-regulated Brd4 transcriptional activities require further investigation, it demonstrates that the oncogenic proline-hydroxylation-dependent pathways may potentially serve as new targets for the development of therapeutic strategy in leukemia.

## MATERIALS AND METHODS

### Cell culture

Hela cell was cultured in Dulbecco's Modified Eagle Medium (DMEM, Fisher Scientific) supplemented with 10% fetal bovine serum (FBS) (Sigma), 100 IU penicillin and 100 μg/ml streptomycin (Fisher Scientific) at 37°C in a 5% CO_2_ incubator. MV 4;11 cell line (a kind gift from Jianjun Chen at the University of Chicago) was maintained in RPMI 1640 media (Fisher scientific) (with 10% FBS and 100 IU penicillin, 100 μg/ml streptomycin) at 37°C in a 5% CO_2_ incubator.

### Generation and validation of pan-hydroxyproline antibody

Randomized hydroxyproline-containing peptide library “CXXXXXX(Hyp)XXXXXX” (“X” refers to any amino acid except for Cys, and “Hyp” indicates trans-4-hydroxyproline) was conjugated to Keyhole Limpet Hemocyanin protein (KLH) and subject to the immunization of rabbit following a standard protocol (SDIX Inc). The antibody was purified with the agarose beads conjugated with hydroxyproline-containing peptide library. For immunoaffinity purification, pan-hydroxyproline antibody was covalently linked to the protein A agarose beads for the immunoaffinity purification using amine-reactive crosslinker as previously described [69].

### Cell lysis and proteolytic digestion for proteomics analysis

Hela cells were lysed on ice in the cell lysis buffer (100 mM NaCl, 20 mM Tris-HCl, 10 mM EDTA, 0.5% (v/v) Nonidet P-40 (NP-40) and 1 mM phenylmethylsulfonyl fluoride (pH 8.0)) followed by sonication. Protein concentration was determined by the Bradford assay (Biorad). For proteolytic digestion, the proteins in the cell lysate were first reduced and alkylated with a final concentration of 5 mM tris(2-carboxyethyl)phosphine (TCEP) and 5 mM iodoacetamide to block cysteine residues. Proteins were then precipitated with 10% trichloroacetic acid (v/v, final concentration), washed three times with cold acetone. The pellet was resuspended in 100 mM NH_4_HCO_3_. The protein solution was adjusted to pH 8.0 and digested by trypsin (Promega) at an enzyme-to-substrate ratio of 1:50 (w/w) at 37°C overnight. The digestion was repeated for the second time for 3 hours at 37°C with an enzyme-to-substrate ratio of 1:100. The digestion was finally quenched by TFA (final concentration 1%, v/v).

### Immunoprecipitation of Hyp-containing peptides

Tryptic peptides were desalted by Sep-Pak C18 column (Waters, 100 mg) following the manufacture's protocol. The eluted peptides were dried by Speed Vac (Thermofisher) and then resuspended in 50 mM NH_4_HCO_3_ buffer. Antibody-bound protein A beads were added to the peptide solution and incubated for overnight at 4°C with rotation. Then the beads were washed twice with the sample buffer and eluted with 0.1% TFA.

### Peptide fractionation and LC-MS/MS analysis

The peptides were fractionated and desalted by Stage-tips packed with Empore membranes (3M) as previously described [70]. Briefly, the peptides were reconstituted in 5% ACN, 0.1% FA (formic acid) in water and loaded onto a Stage-tip packed with Empore Cation Exchange-SR membranes (3M). The Stage-tip was washed with 0.1% FA and sequentially eluted with the following six buffers that contain 0.1% FA (v/v), 20% ACN (v/v) and NH_4_OAc with a respective concentration of 50, 75, 125, 200, 300, 500 mM. Peptides in each fraction were subsequently desalted with C18 Stage-tips and dried in Speed-Vac (Thermofisher). For LC-MS/MS analysis, each sample was resolubilized in 2.5 μl of HPLC buffer A (0.1% formic acid in water, v/v) and loaded onto an in-house packed C18 column (15 cm × 75 μm, ReproSil-Pur Basic C18, 2.5 μm, Dr. Maisch GmbH) through a Proxeon Easy nLC 1000 Nano-UPLC system connecting on-line to an Orbitrap Fusion mass spectrometer (ThermoFisher). Peptides were eluted with a gradient of 5-30% of HPLC buffer B (0.1% formic acid in acetonitrile, v/v) at a flow rate of 200 nl/min. Full mass spectra were acquired with a resolution of 120,000 at m/z 200. Dynamic exclusion was enabled to allow for one fragmentation in 60 seconds. Isolation window for MS/MS analysis was 1.2 m/z. Precursor ions were fragmented using high-energy collision dissociation (HCD) with 35% collision energy in ion trap.

MaxQuant 1.5.2.8 was used to identify peptides from spectra in the raw data. Hydroxylation on proline (+15.9949 Da), protein N-terminal acetylation and methionine oxidation were specified as variable modifications. Carbamidomethylation of cysteine was specified as a fixed modification. Trypsin was specified as the proteolytic enzyme with a maximum of 2 missing cleavages. *In silico* identification of co-eluting peptides from the same spectra was disabled. The precursor ion tolerance was 4.5 ppm and the fragment ion mass tolerance was 0.5 Da. The database search was performed against UniProt human database (downloaded at 2014/04/14 with a total of 69078 sequences) with a cutoff threshold at 1% False Discovery Rate (FDR) at peptide, protein and site levels. Modified peptides were required to have a minimum Andromeda score of 40.

To ensure high confidence identification of hydroxyproline peptides and avoid false identification of peptides containing neighboring Met and Trp oxidation due to ambiguous localization of the modification sites, we applied an in-house developed software script to analyze the fragmentation match pattern of each hydroxyproline peptide identification. For confident localization, the software requires a minimum number of two non-redundant ions in MS/MS spectra that represent peptide fragments between Pro and the closest Met or Trp on both N- and C- terminal side of each peptide. The minimum of two non-redundant fragment ions was specified because the closest possible position of Pro with Met and Trp in the same peptide is that they are next to each other, in which case, only two ions (the b and y ions that fragment between them) can specifically determine whether the mass shift can be localized on Pro or the neighboring Met/Trp residues. The specification of the minimum of two non-redundant fragment ions for the site localization enabled the removal of potentially false positive identifications with very high stringency. All peptide identifications that passed through the fragmentation pattern analysis were further evaluated manually to ensure high confidence of the peptide-spectrum matches. LCMS data are publically accessible through ProteomeXchange Consortium and PRIDE partner repository (accession: PXD005018).

To estimate site-specific Hyp stoichiometry from the analysis of Hyp identifications without affinity enrichment, all the peptide forms containing the Hyp sites were analyzed based on Maxquant search results. The total intensities of modified and their corresponding unmodified peptide forms were summed up respectively to calculate site-specific Hyp stoichiometries.

### Bioinformatic analysis

The pathway and functional annotation enrichment analysis for proline hydroxylation proteome was performed in GOstats software package [71] in R using hypergeometric test for Gene Ontology annotations [72]. The enrichment with a Benjamini-Hochberg [73] corrected P<0.05 was considered to be statistically significant.

Protein-protein interactions were extracted from STRING database [74] with a cutoff interaction score above 700. Protein interaction network was visualized with Cytoscape [75] and highly interconnected protein clusters were identified by MCODE plugin [76]. Human protein complexes were extracted from CORUM database [77] and significantly enriched proline hydroxylation complexes were identified with hypergeometric test with P>0.05.

Protein secondary structure information were manually curated from the PDB data deposited in the UniProt database [78]. Individual protein structures were visualized by Pymol (The PyMOL Molecular Graphics System, Version 1.8 Schrödinger, LLC) with PyTMs plugin [79].

### Synthetic peptide validation

Synthetic peptides were acquired from GL Biochem Ltd (Shanghai, China). The peptides were reconstituted with HPLC buffer A and analyzed by Proxeon nanoLC-Orbitrap Fusion mass spectrometer as described above. Mass-to-charge ratios (m/z ratios) of each peptide based on the endogenous peptide identifications from HeLa cells were specified to acquire targeted HCD MS/MS spectra in ion trap. The data was analyzed by MaxQuant to confirm the peptide identification and evaluated manually for validation.

### Targeted quantification of Brd4 proline hydroxylation

MV4;11 cells were plated at 2×10^4^-2×10^5^/mL and treated for 24 h with 800μM DMOG (Cayman Chemical Company). The cells were lysed in urea lysis buffer (2 mM HEPES pH 8.0, 9.0 M Urea, 12.5 mM EDTA, and 1× HALT protease inhibitor cocktail (Fisher)) followed by sonication. Cysteine was reduced with 5 mM tris(2-carboxyethyl) phosphine (TCEP) (Sigma) and alkylated with 5 mM iodoacetamide (Sigma). The lysate was diluted to 1M urea with 50 mM NH_4_HCO_3_ and adjusted to pH 8.0. The protein solution was digested by trypsin (1:50) overnight, and by a second batch of trypsin (1:100) for additional two hours. The digestion was finally quenched with TFA (final concentration of 1%, v/v).

The tryptic peptides from both control and DMOG-treated cells were analyzed by LCMS as previously described. The precursor ions with the mass-to-charge ratios of 668.3604 and 663.0288, corresponding to hydroxylated and unmodified Brd4 peptides, respectively, were targeted for fragmentation. The mass spectrometry data were acquired with Orbitrap in high resolution for both MS and MS/MS analysis. Specific HPLC elution peaks for each peptide were determined with Parallel Reaction Monitoring (PRM) of multiple product ions [80]. The peak areas of the precursor ions were calculated to determine the abundance of modified and unmodified peptide isoforms. The stoichiometry was estimated with the following formula: Hyp% = (Peak area of Hyp peptide) / (Peak area of Hyp peptide + Peak area of Pro peptide).

### Cell proliferation assay

MV4;11 cells were cultured in RPMI 1640 media with L-glutamine (Fisher) supplemented with 10% FBS (Sigma), 100IU penicillin and 100 μg/ml streptomycin (Fisher). Cells were split at an initial density of 2×10^4^-2×10^5^/mL for proliferation analysis. The chemical treatment began after 24 hours with DMOG or IOX2 (ApexBio). The cell densities were determined every 24 hours in triplicate with a hemacytometer (Spencer). Hela cell proliferation was measured by MTT assay (Sigma) following the manufacture's instruction.

### Western blotting analysis

Proteins were resolved on SDS-PAGE gel and immunoblotted to PVDF membrane (BioRad). Bd4, c-Myc, actin, Hif1α were detected by anti-Brd4 (GeneTex), anti-c-Myc (Cell Signaling), anti-actin (VWR), anti-Hif1α (Millipore) antibodies following the manufacturer's instructions.

Hela cells blank or treated with 200 μM CoCl_2_ for 16 hours were harvested using 2X SDS lysis buffer (125 mM Tris-HCl pH 6.8, 4% SDS, 20% glycerol, 5% β-mercaptoethanol). For the competition assay with the pan-anti Hyp antibody, equal amounts of the Hela whole cell lysate were loaded onto two lanes in gel and transferred onto a PVDF membrane. The membrane was blocked with 3% BSA in TBST (0.1% tween 20, in 1X tris-buffered saline, pH 7.6) for one hour at room temperature. Prior to the incubation with the membrane, the pan-anti Hyp antibody was diluted to a final concentration of 2 μg/ml in 1.5% (w/v) BSA and incubated with 1 μg/ml Hyp- or Pro-containing peptide libraries for 16 hours at 4°C. Then, the antibody solutions were incubated with the membrane at 4°C overnight. The secondary antibody (1:20000) was dissolved in 3% BSA and incubated with the membrane at the room temperature for one hour.

Dot blot assay was performed as previously described [81]. Briefly, peptides in the amounts of 0.125 μg, 0.5 μg and 2 μg were dotted onto the nitrocellulose membrane and dried in air. The membrane was blocked with 5% milk at the room temperature for 1 hour. The pan-anti Hyp antibody was diluted in 2% BSA for a final concentration of 2 μg/ml and incubated with the membrane at the room temperature for 2 hours followed by the secondary antibody incubation. The dot blot was visualized with the VisiGio Prime HRP kit (Amresco).

### Quantitative real-time PCR

MV4;11 cells were treated with 800 μM DMOG for 24 hours with an initial cell density of at 2×10^6^/mL. Control and DMOG-treated MV4;11 cells were subject to qRT-PCR analysis (ARQGenetics Inc., Bastrop, TX). To quantify the levels of c-Myc, Ran, and Rad21 transcripts in MV4;11, standard curves were generated using serial dilutions of each target transcript. The actin was used as the reference gene for normalization. All reactions were run in triplicate. The primers for qRT-PCR are listed in the Supplementary Information (Supplementary Table S5).

### Generation of stable MV-4;11 cell line with HIF-1α knockdown

The pLKO.1-puro vector encoding shRNA targeting Hif1α was purchased from Sigma (TRCN 0000003810*).* Lentivirus for Hif1α knockdown was packaged by the Minnesota Obesity Center (University of Minnesota, MN). To generate the stable knockdown cell line, MV4;11 cells were infected with the lentivirus for four hours in the presence of polybrene (Sigma). Stable transfectants were selected in an RPMI medium containing 10 μg/ml puromycin (EMD Millipore), and about 10^4^ puromycin-resistant cells were pooled and expanded. Knockdown of Hif1α in the stable MV4;11 cells were validated by Western blotting.

### Transient transfection of leukemia cells with Brd4 WT, mutant plasmids and shRNA

The pFlag-CMV2-Brd4 (1-1362) plasmid was a gift from Eric Verdin (Addgene plasmid # 22304) [82]. Brd4 plasmid with P536A mutation was obtained using QuikChange Lightning Site-Directed Mutagenesis Kit (Agilent) following the manufacturer's instructions. The primers are: 5′- GCCCCAGCAGAACAAACGCAAGAAAAAGGAGAAAGAC -3′ (forward) and 5′- GTCTTTCTCCTTTTTCTTGGCTTTGTTCTGCTGGGGC -3′ (reverse). The Brd4 shRNA targeting 3′-UTR (sequence: CCGCCAAATGTCTACACAGTA) was provided by the Genomics Center (University of Minnesota). Transient transfection of MV4;11 cells was performed with the Neon Transfection System (Thermofisher) following the manufacturer's instructions.

### ChIP-qPCR

MV-4;11 cells were washed with PBS and crosslinked in 1% formaldehyde (Sigma) for 10 min, followed by quench with 2.5 M glycine for 5 minutes at room temperature. The cells were washed with PBS, resuspended in cell lysis buffer (10 mM NaCl, 10 mM Tris-HCl (pH 8), 0.4% NP-40, and protease inhibitors (Sigma)) and incubated on ice for 10 minutes. The lysate was centrifuged at 800 g for 10 min. The pellet was washed with PBS and resuspended with 1 mL buffer containing 10 mM EDTA, 0.5 mM EGTA, 10 mM HEPES, and 0.25% Triton X-100 for 10 minutes on ice. After centrifugation, the pellet was resuspended with SDS lysis buffer containing PMSF and incubated on ice for 30 minutes. The lysate was sonicated at 25% power for 10 cycles of 10 sec on and 30 sec off (Sonic Dismembrator Model 500, Thermofisher). After centrifugation, the supernatant was diluted to less than 0.1% SDS with ChIP buffer (150mM NaCl, 50mM Tris-HCl (pH 7.5), 5mM EDTA, 0.5% NP-40, 1% Triton X-100, and protease inhibitors). Four μg ChIP grade BRD4 antibody (Abcam) was incubated with the sample and rotated overnight at 4°C. After clearing the chromatin by centrifugation, the chromatin was incubated with 40 ul (25% slurry) of Protein A/G agarose beads (Santa Cruz) by rotation at 4°C for one hour. The beads were washed three times with cold ChIP buffer. To isolate the input DNA, the input lysate was incubated with 2.5-3x volume of ethanol for precipitation. This sample was washed with 70% ethanol and dried. To isolate input and immunoprecipitated DNA, 10% (wt/vol) Chelex 100 slurry (Bio-Rad) was added to dried pellets or washed beads [83, 84]. The sample was boiled for 10 minutes and then incubated with 20 ug Proteinase K (Sigma) at 55°C for 30min. The digested sample was boiled again for 10 minutes and the supernatant was transferred to a new tube. The input DNA, immunoprecipitated DNA and Protein A/G-only immunoprecipitated DNA were subjected to real time PCR (primer forward: TACTCACAGGACAAGGATGCGGTT, primer reverse: TGAATTAACTACGCGCGCCTACCA [85] (Supplementary Table S5))

## SUPPLEMENTARY FIGURES AND TABLES












