# Supplementary material for: Proteomic analysis reveals diverse proline hydroxylation-mediated oxygen-sensing cellular pathways in cancer cells
Source: Oncotarget. 2016 Oct 13;7(48):79154–69. doi: 10.18632/oncotarget.12632 (PMC5346705; doi:10.18632/oncotarget.12632)
Supplement: Supplementary file 1 [file oncotarget-07-79154-s001.pdf]

Proteomic analysis reveals diverse proline hydroxylation-mediated oxygen-sensing cellular pathways in cancer cells

SUPPLEMENTARY FIGURES AND TABLES

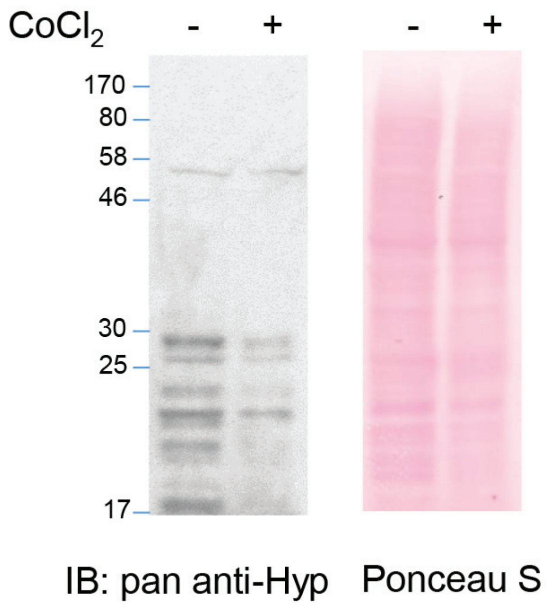

Supplementary Figure S1: Western blotting with the pan anti-Hyp antibody comparing the untreated Hela cells (in Figure 1B) and the cells treated with 200 μM $CoCl_2$, a hypoxia-mimic chemical, for 16 hours.

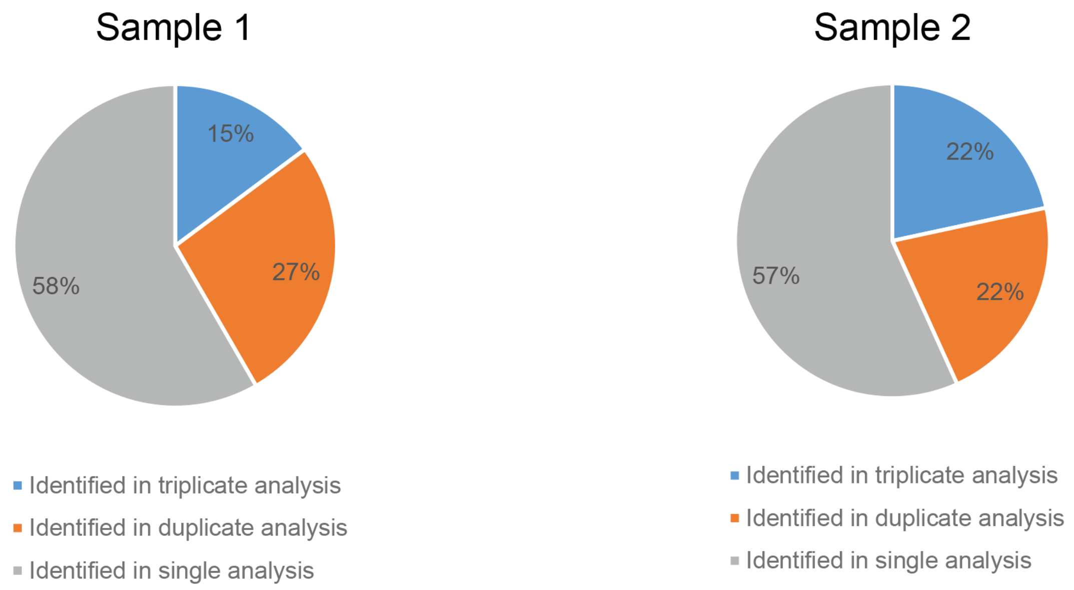

Supplementary Figure S2: Pie charts for the replicate analysis of Hyp proteome with affinity enrichment. The Hyp sites identified in sample 1 and 2 were grouped by the identifications in triplicate analysis (blue color), duplicate analysis (orange color) and single analysis (grey color).

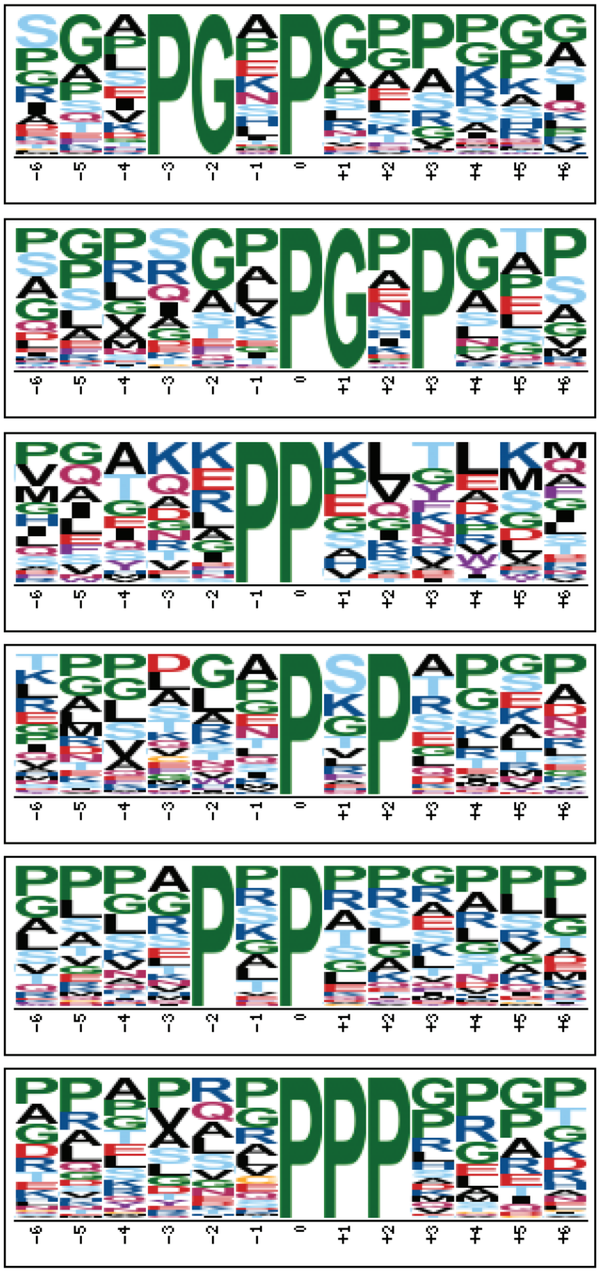

Supplementary Figure S3: Motif enrichment analysis of proline hydroxylation sites identified in this study. Flanking sequence motifs were identified with motif-x program[14] with Bonferroni corrected $P<0.05$.

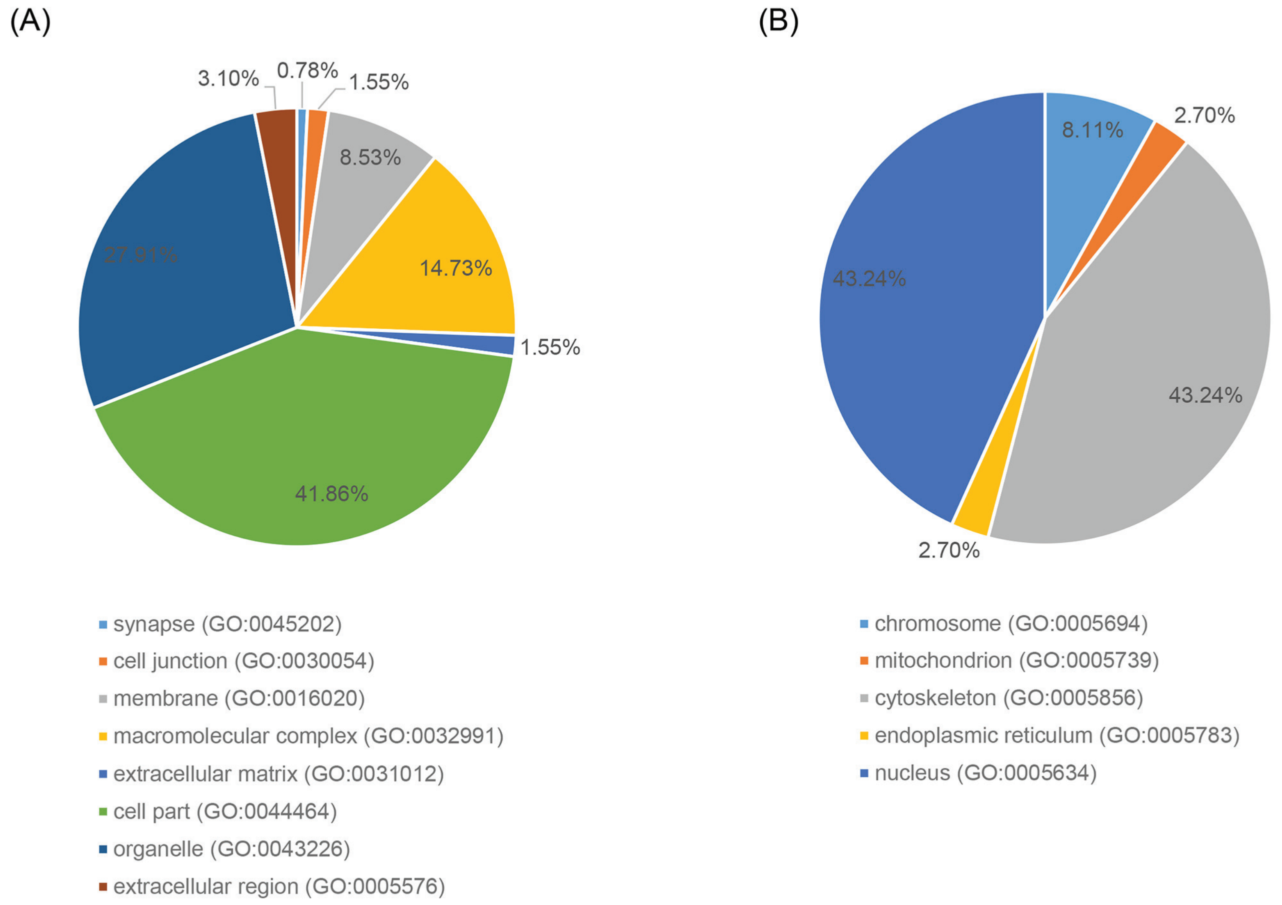

Supplementary Figure S4: Gene classification annotation of Hyp proteome in Hela cells with PANTHER system. **A.** Classification of the Hyp proteome based on the Gene Ontology cellular compartment annotations and **B.** the classification of Hyp proteome that belongs to the category of Organelle (GO:0043226).

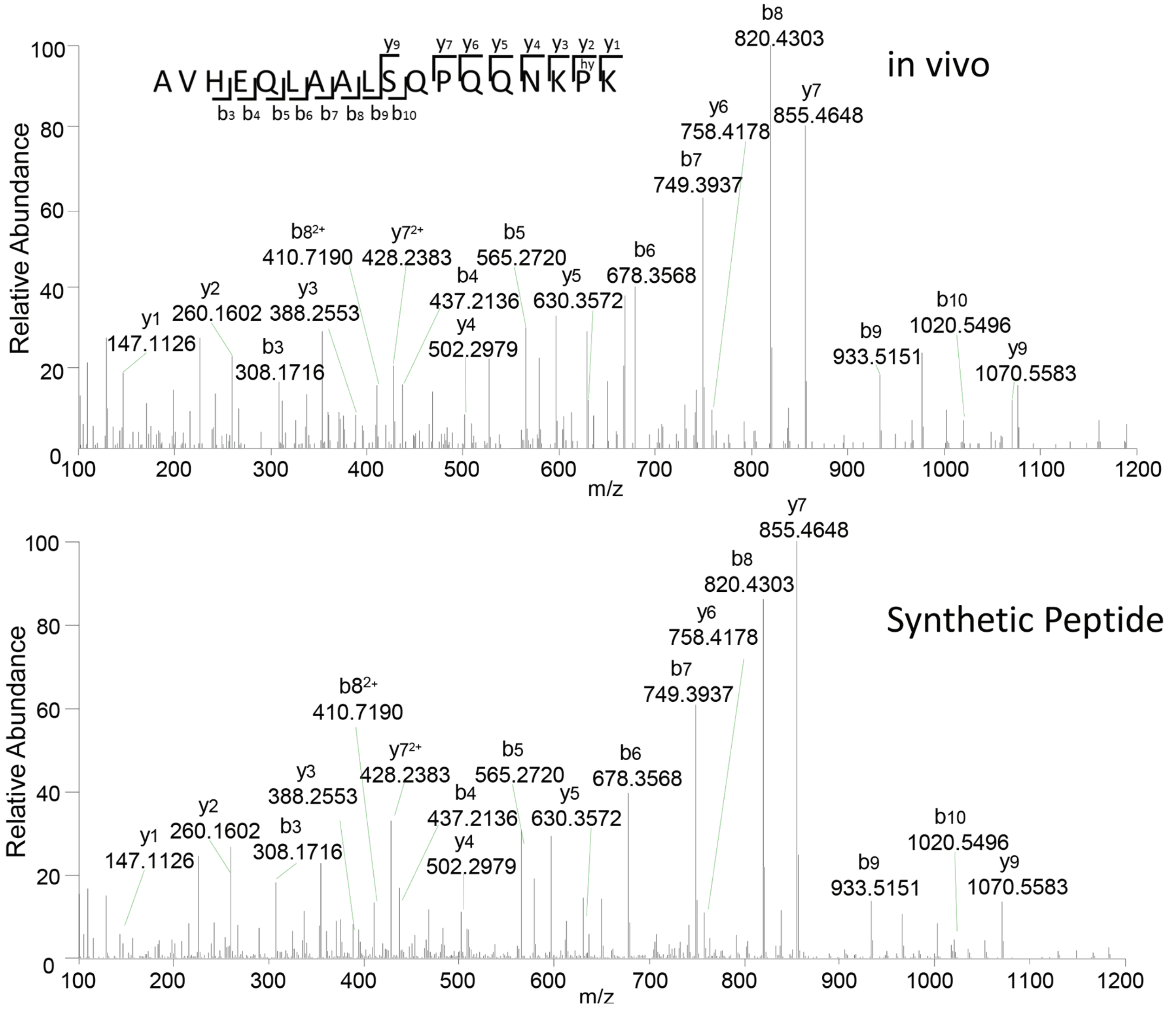

Supplementary Figure S5: Validation of Brd4 Hyp peptide identification with the synthetic peptide analysis and high-resolution HCD fragmentation. "b" and "y" ions designate peptide backbone fragment ions containing peptide N- and C-terminus respectively.

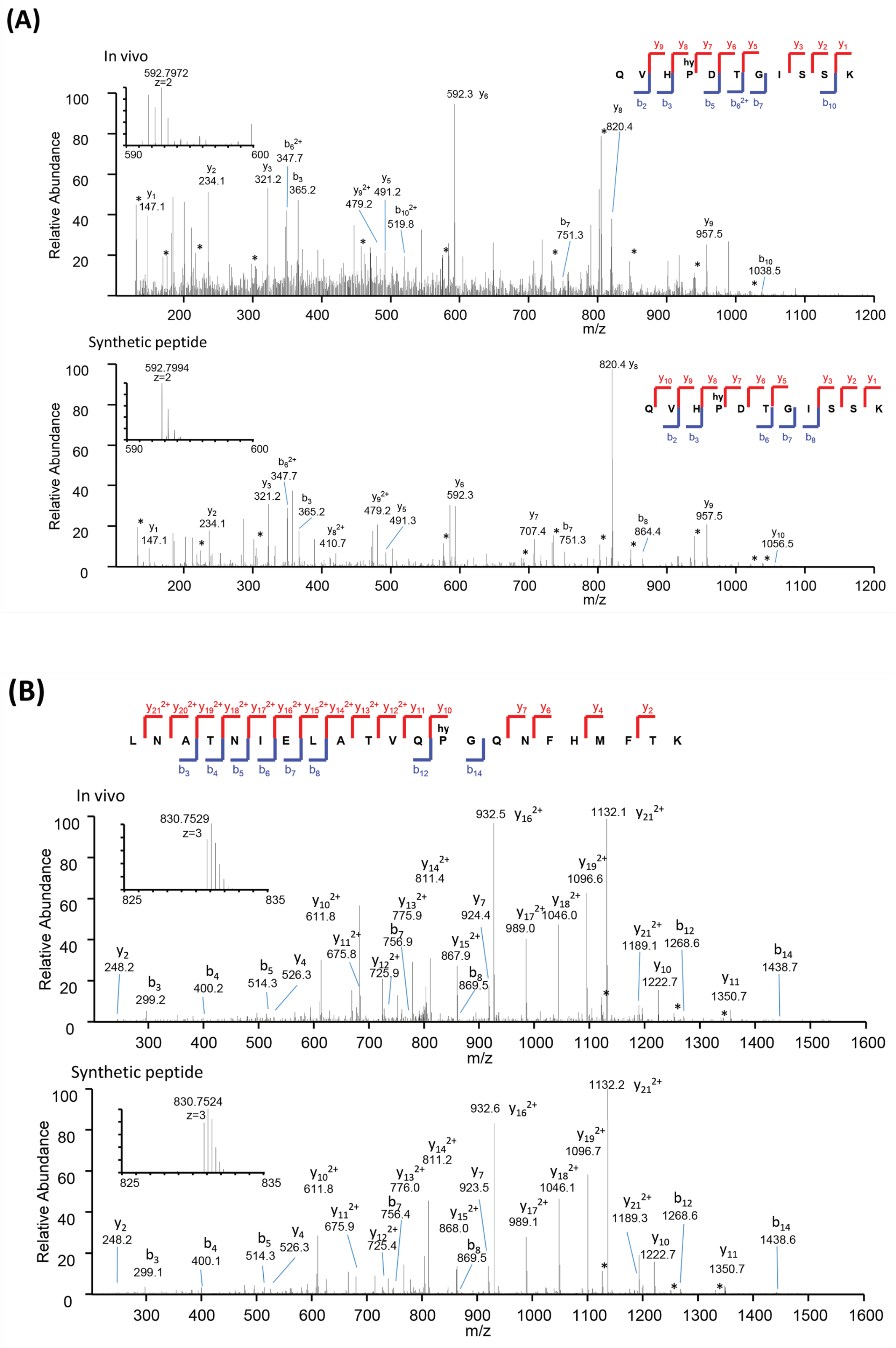

Supplementary Figure S6: Validation of Hyp peptide identification with synthetic peptide CID fragmentation. "b" and "y" ions designate peptide backbone fragment ions containing peptide N- and C-terminus respectively. **A.** Histone H2B type 2-E (UniProt: Q99879) Hyp51. **B.** Proteasome subunit alpha type-5 (UniProt: P28066) Hyp222. (*Continued*)

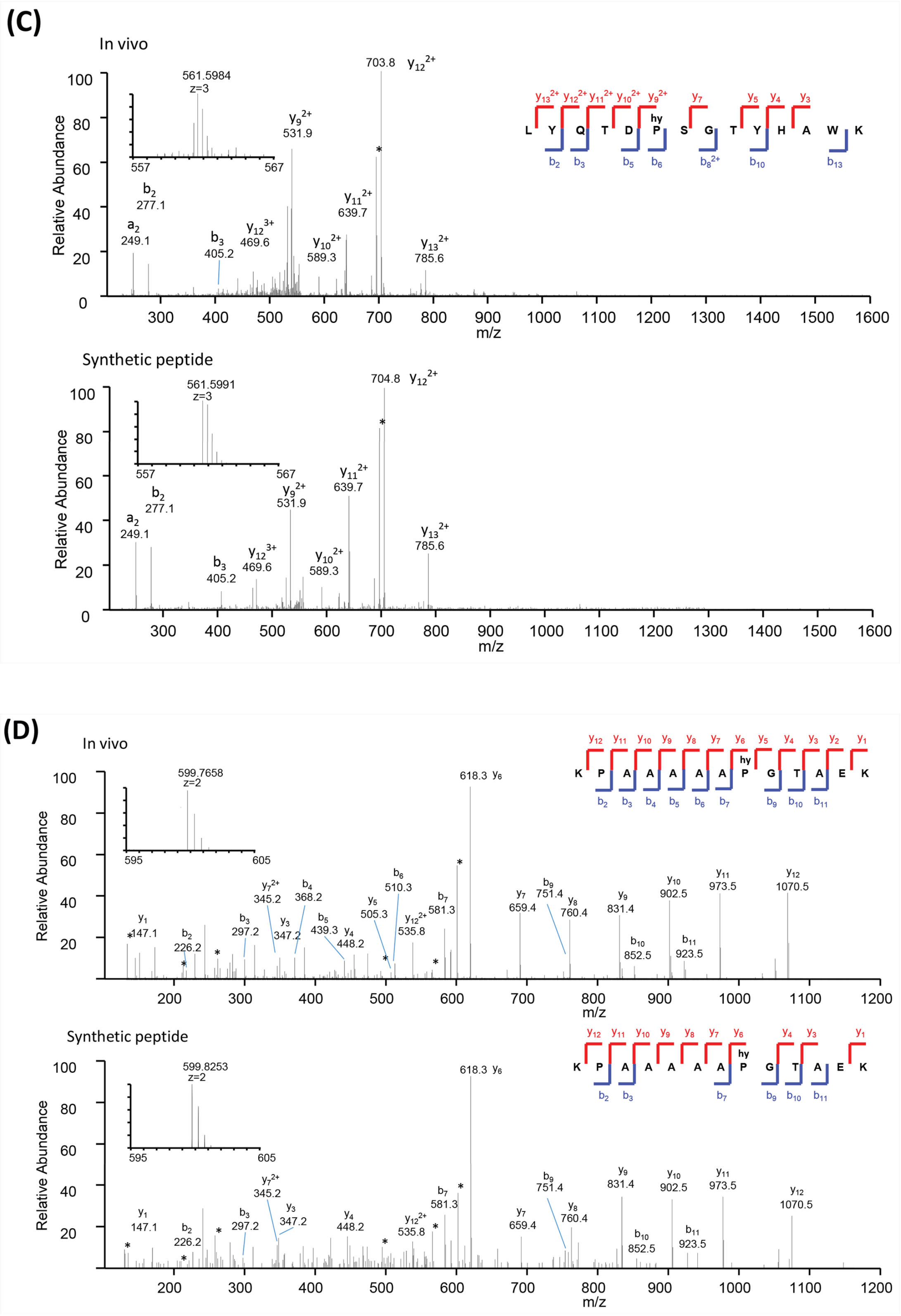

Supplementary Figure S6: Validation of Hyp peptide identification with synthetic peptide CID fragmentation. "b" and "y" ions designate peptide backbone fragment ions containing peptide N- and C-terminus respectively. **C.** Proteasome subunit alpha type-7 (UniProt: O14818) Hyp149. **D.** Serpin H1 (UniProt: P50454) Hyp30. (*Continued*)

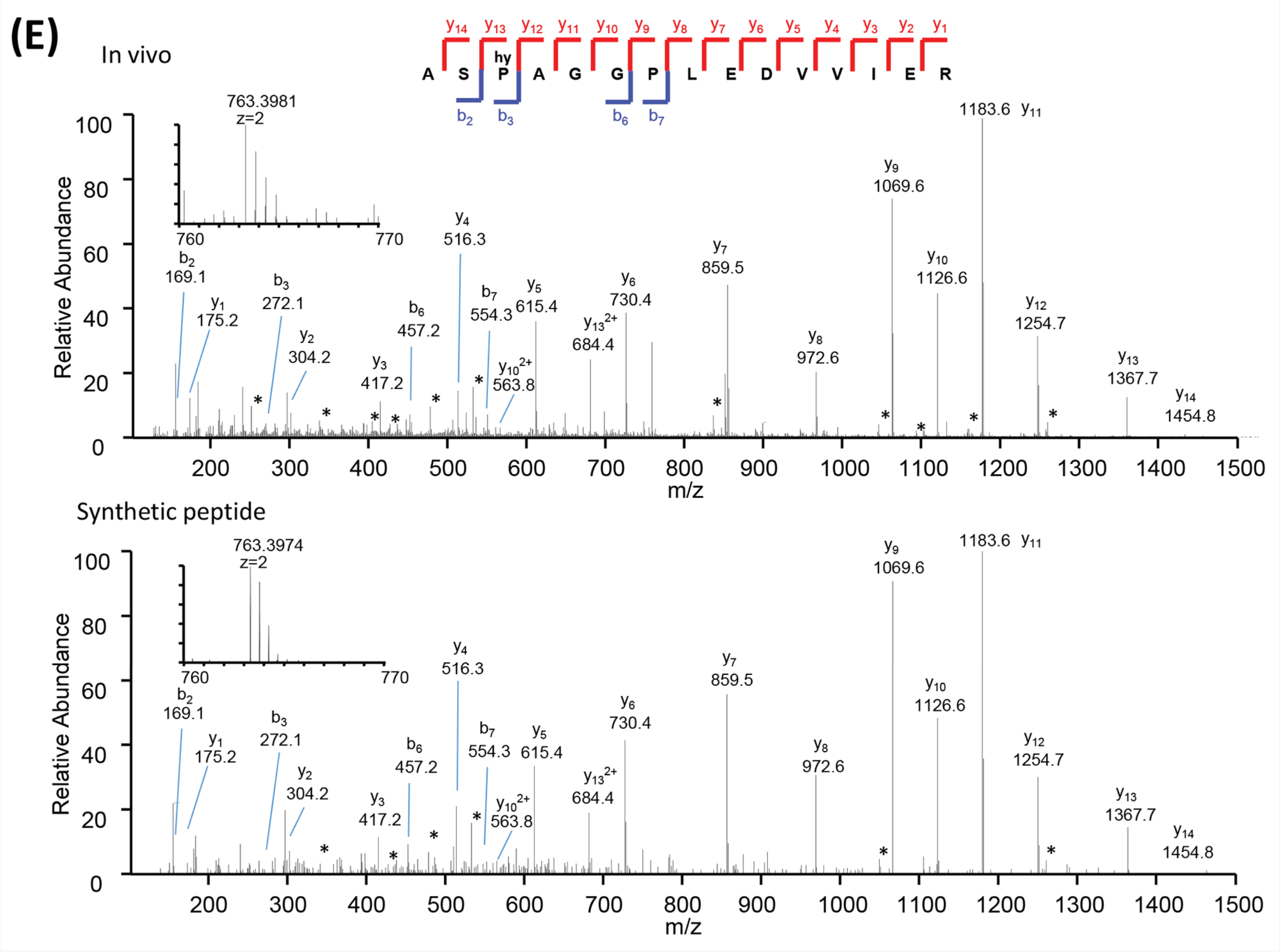

Supplementary Figure S6: (*Continued*) Validation of Hyp peptide identification with synthetic peptide CID fragmentation. "b" and "y" ions designate peptide backbone fragment ions containing peptide N- and C-terminus respectively. **E.** Peptidyl-prolyl cis-trans isomerase FKBP10 (UniProt: Q96AY3) Hyp36.

Supplementary Table S1: The list of proline hydroxylation sites identified in the Hela cells.

See Supplementary File 1

Supplementary Table S2: The list of proline hydroxylation sites with calculated stoichiometries in Hela cells.

See Supplementary File 2

Supplementary Table S3: The enrichment of proline hydroxylation substrates in manually curated CORUM protein complexes.

See Supplementary File 3

Supplementary Table S4: The list of proline hydroxylation proteins involved in stress and shock response pathways.

See Supplementary File 4

Supplementary Table S5: Primers used in qRT-PCR analysis.

See Supplementary File 5
